# Retrieval of spatial representation on network level in hippocampal CA3 accompanied by overexpression and mixture of stored network patterns

**DOI:** 10.1038/s41598-019-47842-w

**Published:** 2019-08-08

**Authors:** Frantisek Zitricky, Karel Jezek

**Affiliations:** Faculty of Medicine in Pilsen, Biomedical Center, Charles University, Pilsen, 32300 Czech Republic

**Keywords:** Cognitive control, Hippocampus

## Abstract

Retrieval of stored network activity pattern has been shown as a competitive transition from one attractor state to another, orchestrated by local theta oscillation. However, the fine nature of this process that is considered as substrate of memory recall is not clear. We found that hippocampal network recall is characterized by hyperactivity in the CA3 place cell population, associated with an “overexpression” of the retrieved network pattern. The overexpression was based on recruitment of cells from the same (recalled) spatial representation with low expected firing probability at the given position. We propose that increased place cell activation during state transitions might facilitate pattern completion towards the retrieved network state and stabilize its expression in the network. Furthermore, we observed frequent mixing of both activity patterns at the temporal level of a single theta cycle. On a sub-theta cycle scale, we found signs of segregation that might correspond to a gamma oscillation patterning, as well as occasional mixing at intervals of less than 5 milliseconds. Such short timescale coactivity might induce plasticity mechanisms, leading to associations across the two originally decorrelated network activity states.

## Introduction

Despite *memory* often being considered as an icon for duration and stability, its processing is very dynamic and in some steps rather elusive. Consequently, while there is vast knowledge about mechanisms of memory encoding, our understanding of its retrieval mechanisms, perhaps for their substantial volatility, seems incomparably poorer.

Physiological substrate of memory is considered as an activity of an ensemble of cells interconnected by a pattern of enhanced synapses in the respective neural networks^1–4^ that behave as distinct states with attractor properties^5,6^. In principle, its recall consists of their reactivation, triggered by a change in the network information input. Such a pattern shift is very volatile, typically taking only a fraction of a second after the occurrence of the respective sensory cue^7,8^. However, its kinetics at the level of neuronal networks is not well understood. Theoretical studies proposed that self-excitation in recurrent networks contributes to the recall and might support it even in the presence of incomplete or altered inputs, in a process called “pattern completion”^5,6,9,10^. In such a situation, the enhanced connections constituting the memory engram can promote its completion after the incoming information activates even only a subset of the original ensemble^5,11,12^. This mechanism ensures that the memory will be retrieved in the reality of constant minor changes of the originally encoded stimuli and faces the fact that even a repetitive experience is never completely the same.

Self-excitation, in addition to other possible mechanisms, such as changes in excitability and short-term plasticity, provides a stabilizing effect on the expressed memory state, causing a hysteretic coding bias in response to a mild change of the input information^13–17^. In this line of thinking, network inertia and input information processing form antagonistic forces whose balance might play an important role in shifting the network memory state during retrieval.

Recently Jezek *et al*.^18^ described the pattern of transition between two hippocampal spatial representations as a response to a sudden change across two familiar environment contexts. They found the transition occurred almost immediately and was often followed by a spontaneous reactivation of the original representation in a competitive, flickering manner. This process was patterned in time by local theta oscillation. To date, we have a very limited understanding about the emergence of the flickering phenomenon. One possibility assumes a conflict between idiothetic- and visual-based inputs^19^. While the visual input is likely to change immediately with the sensory light switch, the self-motion information might take more time to reset. Another possibility reflects the network inertia (including short-term plasticity mechanisms^17^) stabilizing the previous state and alleviating the effect of changed visual information.

Irrespective of which of the two hypotheses better matches the real process, the instability period after the teleportation event provides a unique substrate to assess the mechanisms of network state shift in the hippocampal CA3, a network central to spatial memory formation and retrieval.

In this paper, we provide a deeper insight into the structure of network activity state shift, with a detailed analysis of activity levels, population coding and coactivity across the emerging states. We found that the hippocampal CA3 network becomes considerably hyperactive during the first seconds after its input change. This effect is associated with a selective overexpression of the newly reactivated network pattern. The overexpression is driven by a recruitment of cells from the same spatial representation ensemble, but with a low probability of firing at the given place, affecting in turn the precision of spatial coding in the newly reactivated network state. Finally, we described frequent events of the mixture between the two ensemble patterns, both on theta and gamma oscillation levels.

## Results

**Figure 1:**
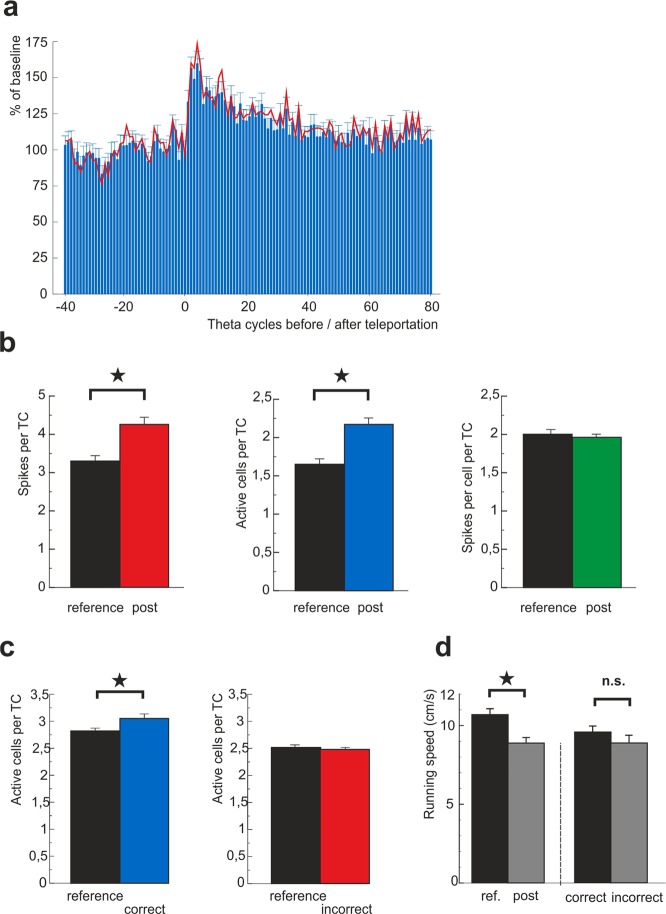
Hyperactivity of hippocampal CA3 network during the network state shift. (**a**) Development of number of active cells per theta cycle in time before and after the teleportation event. The x-axis bins correspond to the individual theta cycles. A cell count within a bin was normalized by the average cell count for all considered pre-teleportation theta bins within the respective recording and averaged across all teleportations. The red line corresponds to spike counts per theta cycle, normalized analogously. (**b**) Average numbers of spikes (left), active cells (middle) and spikes per cell per a theta cycle (right) within 20 theta cycles before and after the teleportation (n = 169). (**c**) Number of cells firing during the post-teleportation “correct” and “incorrect” states and their position-matched controls. The control values were obtained by averaging all the position matched data recorded during the corresponding reference state. There is a significant increase in number of active cells within detected “correct” states but no effect in the “incorrect” ones. (**d**) There was a decrease in the average running speed shortly after the cue switch (left), no significant difference was detected between running speeds during the expression of the “correct” and during the expression of the “incorrect” map (right).

We recorded the activity of 355 hippocampal CA3 pyramidal cells from 6 animals across 11 experimental days (Suppl. Fig. 2). The place cell spiking data from each animal/day was first binned into intervals corresponding to a single theta cycle (TC) derived from the local field activity (6–11 Hz). The border between the bins was set for each individual recording at the phase with the minimum place cell spiking. Initially we ensured the representations for the respective environments replaced each other in response to the sudden environment identity change. We found all teleportations were followed by a categorical change in averaged correlation between the data and the templates corresponding to the environments A and B (Suppl. Fig. 3). Then we compared the spiking activity before and after the teleportation events. We noticed a temporal increase in the number of spikes across the recorded cell population after the environment cues switch. The activity was maximal (peak more than 160% of baseline) within the first 5 theta cycles (corresponding roughly to 600 milliseconds) after the cue switch. The activity then slowly returned to the baseline within approximately 50 theta cycles on average (ca. 6 seconds, Fig. 1a). For a further detailed description, we focused on the first 20 theta cycles (ca. 2.5 sec) of the post-teleportation interval that showed the highest activity increase.

Statistical analysis within the first 20 individual theta bins after teleportation displayed an increased number of spikes (3.30 ± 0.13 spikes per TC pre, 4.25 ± 0.16 spikes per TC post, n = 169 teleportations, Wilcoxon signed-rank test: z = −4.75, p = 2.0307e-06, Fig. 1b). We then asked whether the post-teleportation hyperactivity was caused by an increase in the number of spikes from cells expected to fire at the given location, or by an increase in the total number of active cells. We counted the number of cells that delivered at least one action potential within the given temporal bin. The comparison showed a higher amount of active cells per theta cycle (1.65 ± 0.06 cells per TC pre, 2.17 ± 0.07 cells per TC post, n = 169, Wilcoxon signed-rank test: z = −6.42, p = 1.4048e-10, Fig. 1b), whereas the average number of spikes per active cells per theta cycle remained unchanged (2.00 ± 0.05 spikes/cells per TC pre, 1.96 ± 0.03 spikes/cells per TC post, n = 169, Wilcoxon signed-rank test: z = 0.34, p = 0.7369, Fig. 1b). This shows that the network state recall driven hyperactivity is caused by increased cell population recruitment.

The activity of hippocampal place cells tends to increase with running speed^20^. However, there was an average drop in speed of the rat during the relevant post-teleportation epochs (10.68 ± 0.31 cm/s pre-tele; 8.88 ± 0.32 cm/s post-tele, n = 169 events, Wilcoxon signed-rank test: z = 5.43, p = 5.7652e-08, 2 seconds pre/post, Fig. 1d).

As shown earlier, the network state shift in the hippocampus induced by teleportation is accompanied by occasional flickering between the “new” representation that corresponds to the present visual cues and the “old” spatial representation matching the context shown previously^18^. Because the emergence of the two activity states is supposedly triggered by different mechanisms (the “new” representation (here called “correct”) is directly supported by visual input; the emergence of the “old” representation (here called “incorrect”) is less clear), we decided to analyze them separately.

We first sorted all population activity vectors into predefined categories: “correct” – expressing representation corresponding to the actual present environment – and “incorrect” – data bins corresponding to “flicker events”, expressing representation for the environment present before the light switch (Methods, Suppl. Figs 2,4,5). We took the advantage of a high degree of sparsity and orthogonality of CA3 spatial coding. For each cell, we computed an environment specificity index (*ESI* = *(f*_*A*_ − *f*_*B*_*)*/*(f*_*A*_ + *f*_*B*_), where *f* represents the average activity in Hz for a given cell recorded in environment A or B, respectively), based on the average activity in template sessions across the two environments. The ESI values therefore ranged between −1 and 1 and indicated how each cell activity was specific for each of the two environments (Suppl. Figs 2,4a). The qualitative advantage of this approach compared to the correlation decoder employed in Jezek *et al*.^18^ (2011) is that it is not restricted to the a priori assumption of similarity between the position-specific activity patterns derived from the template conditions and those recorded during the highly dynamic activity evolution after the teleportation event. Instead, such approach is robust to tolerate unpredictable irregularities of population activity within the given network state as e.g. extrapositional firing^21^ within the same global network state that would be undetected under the correlation method used in Jezek *et al*. (2011). Only data bins containing spikes from two or more active cells were considered. The evaluation of the decoder on data from the template session (Suppl. Fig. 4b) confirmed generally high specificity for the decoded map. During the reference epochs of the teleportation session, 59.55% of temporal bins with at least 2 active cells scored as “correct” and 3.04% scored as “incorrect”. Within 20 theta bins after teleportation, the distribution changed to 32.79% for the “correct” and to 12.52% for the “incorrect”, respectively (Suppl. Fig. 4d,e).

Thereafter, we compared the numbers of active cells across all categorized theta bins, with the expected values recorded during the reference conditions at their corresponding locations. We detected an increased number of active cells in theta cycles expressing the “correct” representation (2.82 ± 0.04 cells per theta cycle reference, 3.05 ± 0.07 cells per theta cycle post, n = 377, Wilcoxon signed-rank test: z = 2.80, p = 0.0051), but not in the “incorrect” representation during flicker epochs (2.57 ± 0,04 cells per theta cycle reference; 2.49 ± 0,05 cells per theta cycle post, n = 169 incorrect bins, Wilcoxon signed-rank test: z = −1.42, p = 0.1562, Fig. 1c).

This effect was replicated by a stricter categorization, based on specificity to both position and environment (position specific index, *PSI*, Suppl. Fig. 5a–c), showing a significantly increased number of active cells in bins with the newly activated spatial map (Wilcoxon signed-rank test: z = 3.53, p = 4.1498e-04), but no increase in bins with the “incorrect” map (Wilcoxon signed-rank test: z = 0.82, p = 0.4111, Suppl. Fig. 5d). The running speed showed an insignificant drop during the “incorrect” state expression, while maintaining ongoing theta oscillations (Wilcoxon rank-sum test: z = 0.81, p = 0.42, Fig. 1d).

### Effect of hyperactivity on spatial coding

In the next step, we examined how the detected increase in the activity level of the “correct” representation translates into spatial coding.

The general population vector coding accuracy was estimated by using Pearson momentary correlation between the teleportation session data bins and the corresponding position-matched average activity from the template recordings (as in Jezek *et al*.^18^).

Place cell population activity during theta cycles with the “correct” map displayed lower average correlation with the corresponding template population vector, compared to the environment and position matching control theta cycles at the reference conditions (r_reference_ = 0.66 ± 0.01, r_correct_ = 0.59 ± 0.01, n = 377, paired samples t-test after Fischer z-transform: t = −2.27, p = 0.0237).

**Figure 2:**
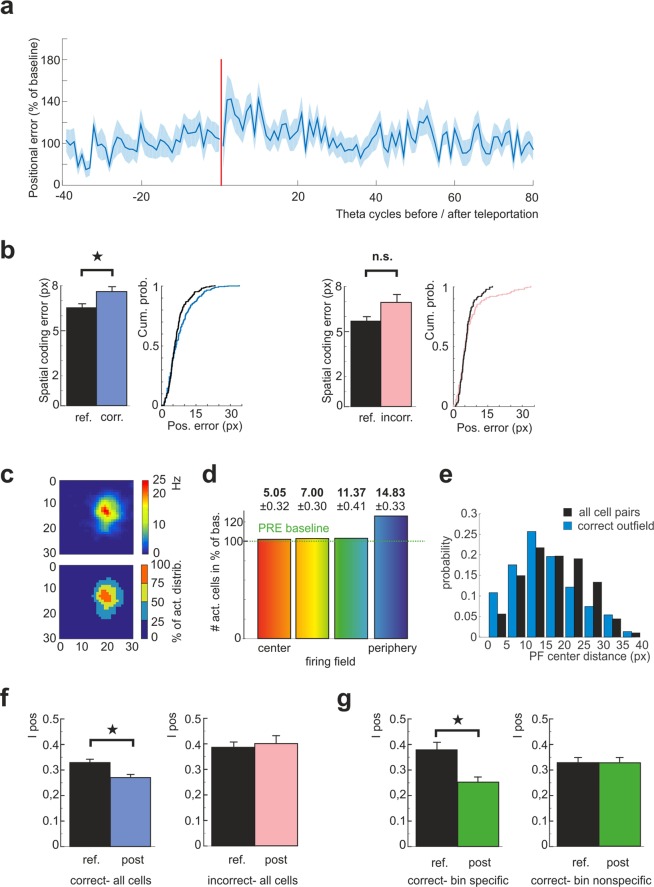
Overexpression of the correct state affects its spatial coding. (**a**) Development of the spatial coding error during the “correct” theta states before and after teleportation. The shaded area shows the corresponding standard error of mean. Baseline represents an average value within the reference period. The post-teleportation period is associated with an increase of the spatial coding error. (**b**) Left: Average coding error during the “correct” (blue) and “incorrect” (pink) states compared with their respective position-matched controls and followed by their corresponding cumulative distribution functions. The significant increase in the coding error was associated with the “correct” but not with the “incorrect” state. (**c**) Example of a place cell rate map (up) divided into four zones (bottom) with borders corresponding to 25^th^, 50^th^ and 75^th^ percentile of the normalized reference firing rate distribution. (**d**) Relative increase in number of the active cells within individual place field zones. The borders between the zones were set to correspond to 25^th^, 50^th^ and 75^th^ percentile of the normalized reference firing rate distribution for each unit. There is a marked increase in place cell activity in the most peripheral zone. Average distance to firing field center ± S.E.M. is indicated above each bar referring to given zone. (**e**) Distribution of distances between firing field centers for coactive pairs of cells in the zone with the lowest probability of firing during expression of the “correct” activity state compared to firing field center distances for all combinations of cell pairs from the same population. (**f**) The positional information for the active cells during the “correct” (left) and during the “incorrect” (right) states, respectively. (**g**) The decrease in the positional information during the “correct” state expression is specific for the cells that are active in the given bin exclusively in the corresponding environment (left). The cells with nonzero firing in the given bin across both environments did not show any positional information decrease (right).

To quantify this effect, we measured the deviation of a real animal’s position from the coordinate with the highest correlation between the given test population vector and any of the template spatial bin population vectors (30 × 30 binning). There was an approximately 17% higher positional error decoded from post-teleportation theta cycles with the “correct” map than from their position-matched controls recorded during the reference period before the teleportation (6.52 ± 0.21 pixels reference, 7.60 ± 0.29 pixels post, n = 377, Wilcoxon signed-rank test: z = 2.36, p = 0.0182, Fig. 2b). When assessing the evolution of position error in time, we revealed its average peak within the first 10 TCs, followed by a decline to the baseline state within the next 4–5 seconds (Fig. 2a).

The correlation of the population vector analysis performed on “incorrect” theta bins revealed no significant change in correlation with the template population vector (r_reference_ = 0.71 ± 0.02, r_incorrec*t*_ = 0.66 ± 0.02, n = 169, paired samples t-test after Fischer z-transform: t = 0.65, p = 0.5171), and no significant change in spatial coding error obtained by the correlation decoder (5.76 ± 0.25 pixels reference, 7.00 ± 0.51 pixels post, n = 169, Wilcoxon signed-rank test: z = 1.01, p = 0.3141, Fig. 2b).

### Why is the coding less accurate?

The results indicate the retrieved spatial network pattern is characterized by activating a higher number of cells and by decreased spatial coding accuracy. To discern its mechanism, we focused on the characterization of the cells active at the moment of the network state shift. We analyzed place cells’ activity with respect to the animal position within their firing field. In line with the notion that there was an increase in the number of spiking cells rather than a change in the number of spikes emitted by the active cells, we considered the firing of a cell within a theta cycle as a single “activity event”, regardless of the number of spikes occurring within the burst. Each activity event from the given unit was assigned a value corresponding to its normalized template firing rate from the respective position bin.

When comparing activity after teleportation with the reference data, we observed a significant decrease in normalized template firing rate values for activity within the theta cycles with the “correct” map (0.37 ± 0.01 Hz reference, 0.34 ± 0.01 Hz new, n = 377, Wilcoxon signed-rank test: z = 3.05, p = 0.0023). This suggests the hyperactivity in the recalled map is associated with recruiting cells further from their respective place field centers. We next divided cell activity events into four categories, depending on their spatial relation to the firing field’s center of each respective cell (Fig. 2c,d). The distribution of activity in correct post-teleportation theta cycles indicated that most of excess activity fell into the zone most distant from the field center, corresponding to lower than ≈10% of the peak firing rate, i. e. beyond commonly defined borders of a place field (change in distribution outfield/infield reference vs post Chi-Square of goodness-of-fit test: X2 (1, N = 1150) = 1.0967e + 04, p = 0, examples in Suppl. Fig. 6). A similar effect was obtained when considering position bin-specific activity vectors (Suppl. Fig. 5e). In contrast to the place cell activity coding for the “correct” environment, firing during the flickers did not show a deviation from the reference patterns (0.42 ± 0.01 Hz reference, 0.41 ± 0.02 Hz post, n = 169, Wilcoxon signed-rank test: z = −0.87, p = 0.3845).

The compromised spatial firing was further confirmed by calculating positional information - *I*_*pos*_^22^, conveyed by individual activity events. For each active cell within a theta cycle expressing the correct map, we calculated positional information (*I*_*pos*_) for corresponding contexts (Fig. 2f). Estimated *I*_*pos*_ values for post-teleportation activity was lower than in the reference dataset (*I*_*pos*_ reference = 0.33 ± 0.01, *I*_*pos*_ post = 0.27 ± 0.01, n = 377, Wilcoxon signed-rank test: z = −4.17, p = 2.9815e-05). To see whether this effect depended on a cell response specificity, we categorized the cells into highly specific cells (non-zero expected activity at the given position bin only in one environment), and cells with low environment-specific activity levels at the given place. We found the effect was retained in highly contextual specific cells (*I*_*po*s_ reference = 0.38 ± 0.03, *I*_*pos*_ post = 0.25 ± 0.02, n = 230, Wilcoxon signed-rank test: z = −4.49, p = 7.2349e-06), while it was diminished for the cells with the place field covering the respective bin in both environments (*I*_*pos*_ reference = 0.33 ± 0.02, *I*_*po*s_ post = 0.33 ± 0.02,n = 293, Wilcoxon signed-rank test: z = −1.2, p = 0.2265, Fig. 2g).

The *I*_*pos*_ calculated for cells active during the flickers did not show a significant difference between the post-teleportation and the reference conditions (*I*_*pos*_ reference = 0.39 ± 0.02, *I*_*pos*_ post = 0.40 ± 0.03, n = 169, Wilcoxon signed-rank test: z = −0.13, p = 0.8932, Fig. 2f).

The observed augmented place cells’ activity after teleportation can therefore be attributed to increased activity at the periphery of the firing fields, i.e. in the zones with relatively low firing probability during the reference conditions of the given network state expression. To investigate whether the cells displaying the excessive firing at their field periphery differed from the rest of population, we calculated the mean firing rate for given context, ESI and place field size of cell per activity event occurring at the periphery of place field for activity during”correct” post-teleportation theta bins and associated controls. We didn’t find a significant difference in any of the parameters (mean rate: 1.32 ± 0.06 Hz reference, 1.37 ± 0.06 Hz post, Wilcoxon rank-sum test: z = −0.15, p = 0.8784, ESI: 0.71 ± 0.02 reference, 0.69 ± 0.02 post, Wilcoxon rank-sum test: z = −0.51, p = 0.6107, field size: 53.79 ± 2.05 pixels reference, 57.20 ± 1.92 pixels post, Wilcoxon rank-sum test: z = 1.30, p = 0.1929). The distribution of place field center distances of cells coactive at periphery of their place fields during single theta cycle displayed shift to shorter distances compared to all combinations of cell pairs from the same population (Kolmogorov-Smirnov test: p = 7.5233e-07, Fig. 2e).

### Mixed cycles

**Figure 3:**
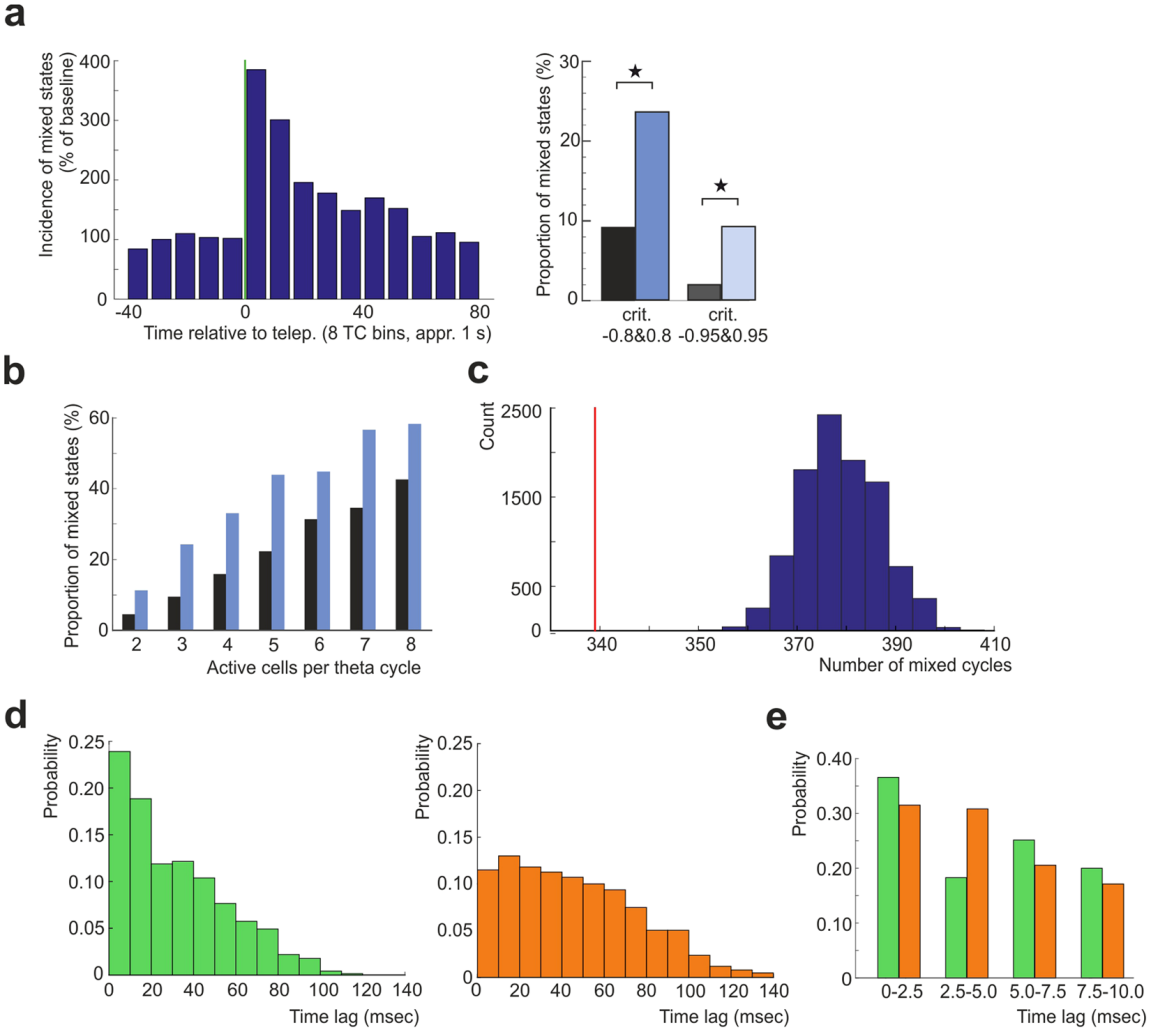
Mixed representations occur across theta and gamma scales. (**a**) Left: Temporal evolution of the mixed states average incidence before and after the cue switch. Each bin of the histogram corresponds to the average value across eight subsequent theta bins (approximately 1 second). The baseline was set to the average across the pre-teleportation interval. Right: Increase in detected mixed states after teleportation compared to the reference epoch, employing two inclusion criteria of different strictness (black: stable, blue: post-tele, left: ESI > 0.8 & < −0.8 vs right: ESI > 0.95 & < −0.95). (**b**) The proportion of mixed states among all theta cycles containing different number of active units (black bars – stable, blue bars – post-teleportation). The apparent increase of mixed states in all the categories suggests that the higher detection during post-teleportation period is not a statistical side effect of the place cell hyperactivity. **(c**) Incidence of mixed states (red mark) compared to the shuffled data. There was a smaller amount of mixed stated than in 10 000 cases of random shuffling across the post-teleportation population vectors (see Methods). (**d**) Distribution of the time lags between the spikes from cells within (top) and across (bottom) both representations (cutoff |ESI > 0.95|). The place cells from the same representation are more likely to fire together at short timescales, reminiscent of gamma rhythm coordinated cell assemblies. (**e**) Detail of distribution of the cross-correlation time lags within the 0–10 millisecond temporal window from (**d**) between the spikes from cells within (top) and across (bottom) both representations.

A considerable amount of post-teleportation population vectors expressed joint similarity for either of the two template network patterns (Suppl. Fig. 2e). Mixed data bins were therefore defined as theta cycle-based population vectors, with detected activity of at least one pair of cells displaying highly specific firing for mutually different environments (*ESI* > 0.8 & *ESI* < −0.8). We compared the distribution of the mixed cycles after teleportation with that during the reference conditions. The period after the switch of spatial contexts showed an increase in mixed cycles’ incidence from 9.10% (reference) to 23.55% (post) (two-way ANOVA, the main effect of reference/post condition, F(1,6) = 65.91, p = 0.0002). Stricter environment selectivity criteria supported the effect (1.91% (reference) to 9.19% (post) (*ESI* > 0.95 & *ESI* < −0.95, two-way ANOVA, the main effect of reference/post condition: F(1,6) = 34.26, p = 0.0011; the main effect of number of active cells category: F(6,6) = 4.87, p = 0.0377). To account for an eventual binning artifact, we re-binned all the data according to the theta phase, returning the minimal amount of post-teleportation populations’ mixing (9.00% (reference) to 21.56% (post), the main effect of reference/post condition: F(1,6) = 44.17, p = 0.0002; the main effect of number of active cells category: F(6,6) = 10.24, p = 0.0001). The same results were returned when a mixed cycle was defined as coactivity of a cell pair originally firing at a given position bin in mutually exclusive environments (PSI = 1 & PSI = −1, 4.50% (reference), 14.26% (post), two-way ANOVA, the main effect of condition: F(1,6) = 25.52, p = 0.0023; the main effect of number of active cells category: F(6,6) = 10.24, p = 0.0061). The increase in the mixing was apparent across all categories, when dividing the theta population vectors according to the number of active cells (Fig. 3b) (two-way ANOVA, the main effect of reference/post condition: F(1,6) = 65.91, p = 0.0002; the main effect of number of active cells category: F(6,6) = 35.09, p = 0.0002). The theta amplitude during mixed states did not differ significantly to theta amplitude during “correct” and”incorrect” states (ANOVA: F(2,8) = 0.53, p = 0.5941).

Despite the incidence of pattern mixing after teleportation being considerably high, the observed number of mixed states was however significantly lower than in the shuffled data obtained by random permutations of cell activity across post-teleportation population vectors (p < 0.0001, Fig. 3c), replicating the previous finding of attractor organization of network activity^18^.

We next asked whether the mixing of the ensemble activity could be detected even at shorter timescales. Examination of interspike intervals between the activity of the highly specific subpopulation of cells (abs(*ESI*) > 0.95) within and across the two representations during the detected mixed states (Fig. 3d) revealed a prominent coactivity in the 10 msec lag for cells specific to the same ensemble. For the cells specific to the concurrent ensembles such coactivity was less prominent but still present. To assess whether the post-teleportation retrieval period was associated with the increased incidence of such coactivity at short timescales, we recut the data into 10 msec temporal bins and found an increased incidence of mixed bins in post-teleportation interval (0.04 ± 0.02 bins/tele pre, 0.33 ± 0.08 bins/tele post, n = 169, Wilcoxon signed-rank test: z = −4.14, p = 3.4843 e-05). Activity at such a short timescale is organized by gamma oscillations (25–100 Hz) and these results indicate a possible coactivity at the single gamma cycle. To support this assumption we binned the data based on LFP filtered at gamma frequency range (25–100 Hz). We confirmed the increased emergence of mixing after the teleportation compared to the reference period (0.05 ± 0.03 bins/tele pre, 0.49 ± 0.11 bins/tele post, n = 169, Wilcoxon signed-rank test: z = −4.71, p = 2.5337e-06). Finally, a detailed examination of the cross-correlation time lags inside the 0–10 msec window showed a peak close to zero lag, both within and across the ensembles, however with stronger coactivity for the peer neurons (Fig. 3e). The coactivation of the orthogonal patterns thus occurred even at short timescales relevant for cell assembly dynamics and synaptic plasticity.

### Local field potential and interneurons

The increase in population activity within and across the spatial representations could be potentially related to changes in network oscillations. We tested whether the theta rhythmicity was affected after the teleportation event in comparison to the pre-teleportation reference state. The averaged time-frequency representation across all teleportation events from the individual animals showed a marked increase in theta amplitude within approximately two seconds after the teleportation (Suppl. Fig. 7a). This effect was quantified by averaging the theta amplitude across the two second interval before and after the teleportation event, respectively. We found the theta amplitude was significantly higher after the light switch than the corresponding reference values (z-score theta amplitude: pre: 0.07 ± 0.03, post: 0.38 ± 0.04, n = 169, Wilcoxon signed-rank test: z = −4.59, p = 4.3773e-11). In contrast, no effect was seen in the duration of the theta wave (n = 169, 0.12 ± 0.00 s pre, 0.12 ± 0.0 s post, Wilcoxon signed-rank test: z = 0.38, p = 0.7040, Suppl. Fig. 7b–d).

Sixteen interneurons were recorded simultaneously with pyramidal cells on some of the tetrodes (Suppl. Fig. 8). The analysis of their activity showed a dual response in respect to the teleportation event. A minority expressed an activity drop, whereas most of them returned an increase in firing frequency during the post-teleportation period (examples in Suppl. Fig. 8a–c,f). To assess their response in general, we z-scored their activity evolution within the interval of 50 theta cycles before and after the light switch and averaged their absolute z-score values across the interval of 2 seconds before and after the teleportation, respectively. The absolute value ensured we could detect an eventual activity change independently of its polarity. The absolute z-score was significantly higher after the environment identity change than before (Wilcoxon signed-rank test: z = −3.52, p < 0.01, Suppl. Fig. 8d,e).

### Dynamics of network state stabilization

We observed a high variability in expression of the “correct” and “incorrect” states during the examined post-teleportation periods on the individual recording days (Suppl. Tab. 1). We speculated that this might reflect variability in the course of network state stabilization, supposedly reflecting the different relative strength of entrainment of both representations during the conflict periods. We thus decided to examine the data separately from the high-flickering days (number of “incorrect” >50% of number of “correct”) and low-flickering days (number of “incorrect” <30% number of “correct”). We noticed stronger activation of place cells during the correct states on the low-flickering days (2.4 ± 0.05 cells per theta cycle reference; 3.25 ± 0.11 cells per theta cycle post-tele, Wilcoxon signed-rank test: z = 4.64, p < 0.00001, n = 208), but not on the high-flickering days (2.70 ± 0.08 cells per theta cycle reference, 2.63 ± 0.09 cells per theta cycle post-tele, n = 114, Wilcoxon signed-rank test: z = −1.08, p = 0.2787). This suggests that hyperactivity might support the corresponding state (i.e. the correct one) in the competition that follows the sensory input switch.

## Discussion

In this study, we provided quantitative characteristics of the transition between two network activity patterns in hippocampal network CA3. We found the competitive process of state transition was accompanied by marked network hyperactivity. Secondly, we identified the two patterns occasionally mixed at sub-theta temporal levels. The ensemble activity state shift was induced by what is called a “teleportation event” – a sudden change of intra-maze light cues that controlled the environment identity^18^. As shown before, a teleportation event is often followed by a period of network state competition, manifested by sequential expression of both the newly activated (“correct”) and former (“incorrect”) activity patterns. This transitory condition enables an effective insight into the fine kinetics of a spatial map recall processing on the network level. The network hyperactivity described here emerged immediately after the sensory shift and lasted on average for up to a couple of seconds. The effect was driven by temporary recruitment of a higher number of cells per time unit, whereas the average level of spiking per an active cell was kept unchanged. The excess of activity was rather strong, peaking on average at almost 160% of the baseline. Further analysis using theta bin categorization has shown that the hyperactivity phenomenon was specific to the reactivated network pattern (“correct”), because the level of activity during the transiently emerging previous state (“incorrect”, flickering) was lower or unchanged (depending on the criteria used). We applied two independent decoding approaches with various criteria for categorizing the data into either activity state (“correct” vs. “incorrect” and “mixed”) with consistent results. Next, we proved that the excessive cell population came from the same hippocampal spatial ensemble as if the reactivated network state had become overexpressed. The recruited cell subpopulation had a low probability of firing at the given place, as their place field centers were more distant from the current animal position. From this point of view such a case might resemble the theta assemblies, coding for the possible future paths within the active network state and therefore reactivating cells with firing field centers distant from the actual position of the animal^23^.

What is the origin, the mechanism and the presumed role of the transient overexpression of retrieved network state in CA3? Intuitively, a sudden change in the light cues driving the environment visual identity was likely to trigger an alertness response. Behaviorally, the teleportation event was followed on average by a small drop in the running speed, while theta rhythmicity was maintained. Often the animal did not even show any signs of behavioral response, neither orientation nor speed decrease. Nevertheless, should a general arousal be causing the hyperactivity effect, we would rather expect it across both correct and incorrect states. However, in the data only the newly reactivated pattern was overexpressed. Another (to our view more likely) possibility to consider is a supposed lower level of spike frequency adaptation within the newly recalled ensemble. Together with the environment cue-switch, the cellular ensemble corresponding to the newly recalled state might respond with an increased level of activity to the depolarizing inputs due to a lack of adaptation^24^, while the original network state should not. Such a difference, consistent with the data, would lead to a specific overexpression of the correct ensemble activity pattern. Moreover, one would expect the hyperactivity to be pronounced in cells with an otherwise low probability of firing, because the units with firing fields centered at the current animal location might fire close to their ceiling, regardless of the adaptation level. The data support this, as the altered spatial firing pattern of the reactivated network state shifted the predominant place cells’ activity towards their firing field periphery. Such a change of firing characteristics was reflected in an increase in positional error decoded from the post-teleportation activity vectors. The effect remained robust in a replication when position-specific firing was used for the vector classification, limiting an eventual effect of “contamination” by the alternative map. In addition, we cannot rule out more complex non-trivial phenomena emerging from the interaction between network states triggered by the conflicting inputs.

In addition to the above-mentioned adaptation mechanism, the lower precision of spatial code might arise as well from a mismatch between the two main inputs supporting navigation during the post-teleportation conflict phase. It is assumed that, shortly after the cue switch, visual-driven input triggers activity of the”correct” map, while the path-integrator input might still correspond to the previous network state. This twofold input conflict might be present until a realignment of the grid cell system takes place within a couple of seconds^19,25^. During such a conflict phase, the activity within the correct map is supposedly guided by visual input only, lacking the support from self-motion cues which might decrease the precision of spatial coding^26^. Similarly, the coding accuracy in the “incorrect” population vector bins tended to be decreased too, though not significantly. Such a trend could arise from the inverse situation of isolated path integrator input.

The appropriate grid cells’ realignment might involve the feedback projections from place cells^27,28^ and enhanced expression of the “correct” pattern might therefore facilitate the reset of the path integrator and resolution of the conflict. It is worth noting that the interaction between path integrator and other inputs is an ongoing process required for successful navigation even in a constant environment. As a path integrator by its nature is prone to cumulative errors, it requires a periodic correction to maintain congruence with visual and other distant cue-bound inputs whenever their mismatch occurs^28^. Whether or not the related network activity shows similar dynamics, including the overexpression of the visual-driven ensemble, is unknown. However, selective hyperactivity in the “correcting” pattern during the mismatch might effectively impose the reset of the path integrator by hippocampal feedback on the medial entorhinal cortex.

In the post-teleportation interval, a large number of data bins containing cells specific for either environment were identified, constituting another origin of the hyperactivity phenomenon. Their occurrence persisted, despite the multiple criteria and methods used. A systematic shift of the theta phase used to separate data bins did not eliminate their emergence either. In Jezek *et al*.,^18^ expression of the mixed states detected on the level of theta temporal binning was accentuated in the first portion of the theta cycle, suggesting the network state segregation might occur at a finer temporal (e.g. gamma) scale^8,23^. We analyzed the interspike intervals between cells specific to the non-corresponding representations across different time lags. We showed a lower incidence of across-states, compared to within-state coactivity at short temporal scales (<10 msec)^22^. However, the detected coactivity between cells from different ensembles was still distinct at intervals even shorter than 10 milliseconds, suggesting that distinct spatial maps’ overlap at gamma-level timescale is possible. This was confirmed when cutting data according the local gamma oscilations (25–100 Hz). Such co-firing corresponds to the time window of spike-timing-dependent plasticity^29^. It is therefore possible that it might influence the cross-ensemble segregation and dynamics in the long term, providing an eventual substrate for attractor merging.

The described enhancement of recalled spatial representation could be of general importance in the mechanisms of memory retrieval. In the scenario with two concurrent inputs, each engaging specific ensemble, the attractor-type dynamics reflect even a small difference in pattern expression strengths, so that finally the network can be fully complete into one memory state, while abandoning the other. Due to its nonlinear nature, such an “overexpression”, even of short duration, might significantly shorten the states’ competition in favor of the newly recalled network pattern. From works of others^21,22^ we know that a fast switching between different network states accompanies neural processing of conflicting information inputs or a future decision making. Its occurrence therefore seems to be important for solution of various spatial memory tasks and the overexpression of the newly emerging pattern might play an important role in stabilizing it. Whether the overexpression is limited to CA3 network processing, or whether it propagates down to CA1 and eventually back to the deep layers of entorhinal cortex to facilitate the supposed path integration reset, still remains to be resolved in upcoming studies.

## Methods

### Summary of experimental procedures

All protocols followed in this study were approved by the Ethical Committee of the Ministry of Education, Youth and Sports of the Czech Republic (approval no. MSMT-10669/2016) according to the Guide for the Care and Use of Laboratory Animals (Protection of Animals from Cruelty Law Act No. 246/92, Czech Republic.) Neuronal activity was recorded as described in Jezek *et al*.^18^ In brief, six male Long-Evans rats were implanted with a hyperdrive containing 14 independently movable tetrodes, which were then slowly lowered into the CA3 pyramidal layer. The reference tetrode was positioned into the corpus callosum and another tetrode was positioned in the stratum lacunosum-moleculare for acquisition of reference EEG signal. The rats were trained to develop different place cell maps for two square-shaped (60 cm × 60 cm) environments. The identity of the environment was defined by a specific configuration of light cues present in otherwise identical enclosures. The extra-maze cues were eliminated by dark curtains surrounding the training apparatus. The test protocol consisted of two reference sessions, one for each environment, followed by a teleportation session. In the teleportation session, a rat was initially placed into one of the environments and, after of 40–60 s of foraging, the light cues were switched to the alternative configuration, creating a step-wise change in the context identity. Subsequent “teleportations” occurred every 40–60 s of free exploration. Detailed experimental protocol and recording procedures are described in the Supplementary Material.

### Data analysis

#### Template rate maps’ construction

For each unit, the spatial rate maps for the template sessions were created by binning the surface of the arena into 30 × 30 position bins (2 cm × 2 cm) and calculating the average firing rate, by dividing the number of spikes by the total dwell time for each bin. The maps were further smoothed with Gaussian average over surrounding 2 × 2 bins.

#### Theta cycle population vectors

To analyze activity at the level of individual theta cycles, spike trains from each session were cut into individual theta bins, with a border corresponding to the phase with the lowest average population activity. Theta phase values were extracted from LFP signal bandpassed for theta frequency (6–11 Hz), by interpolating between detected peaks and troughs. The spiking activity per unit falling into a single bin corresponded to the respective population vector.

Each population vector was linked with an instantaneous position of the rat, based on 30 × 30 binning of the arena surface. These spatial coordinates were used for the analysis of spatial coding (see below) and defining position-matched controls, where a match of both coordinates was required.

#### Hippocampal network state shift verification

To verify that cue switch induced transition of spatial representation from the one corresponding to the previous environment to the representation of the current environment, we calculated average correlation of momentary population vector with corresponding template vector for both environments across all theta bins from all inter-teleportation intervals. High correlation for one template and low correlation for the concurrent template indicated expression of the respective place cell map. We confirmed that each teleportation was followed by corresponding network state interchange.

#### Environment specificity index and position specificity index

To classify population vectors according to the expressed place cell ensemble, we constructed an Environment Specificity Index (*ESI*), defined as: *ESI* = (*f*_*A*_ − *f*_*B*_)/(*f*_*A*_ + *f*_*B*_), where *f* represents the template average activity in Hz for a given cell recorded in environment A or B. *ESI* therefore ranged between −1 and 1 and indicated how each cell activity was specific for each of the two environments. The population activity vectors were considered as “correct” if they contained at least one highly specific cell for the currently present environment context (*ESI* >  = 0.8) and no other cell with *ESI* < −0.2. Opposite criteria were set for the “incorrect” activity bins (at least one cell *ESI* < = −0.8, other cell(s) *ESI* < = 0.2). Only theta bins with at least 2 active units were considered. As “mixed” data bins were considered vectors simultaneously expressing cells specific for both contexts (*ESI* > = 0.8 & *ESI* < = −0.8 or *ESI* > = 0.95 & *ESI* < = −0.95 when stricter specificity was desired).

The context-position specificity of firing was assessed by introducing the Position Specificity Index (*PSI*), defined for each cell and position bin as *PSI* = (*f*_*xA*_ − *f*_*xB*_)/(*f*_*xA*_ + *f*_*xB*_), where *f*_*xA*_ and *f*_*xB*_ are the cell’s template firing rates at position bin *x* in the respective environments, corresponding to the values from the smoothed template rate maps. We set *PSI* equal to 1 if a cell displayed absolute specificity for the correct context in the corresponding bin, while *PSI* equals −1 in the case of specificity for the alternative context. The “correct” vectors were then defined as those containing activity of at least two cells with *PSI* = 1 and no active cell with *PSI* = −1, while “incorrect” met the opposite criteria (2 cells with *PSI* = −1, no cell with *PSI* = 1).

#### Post-teleportation and reference period

The post-teleportation period included a full 20 theta bins following the cue switch. The reference period, framing the control data, corresponded to the time interval between the individual teleportations, excluding 80 theta bins following each cue switch. For some analyses, the activity directly preceding the teleportation was used as a control.

#### Quantifying hyperactivity

Active cell counts during theta cycles expressing the “correct” and “incorrect” (flickers) momentary network states (detected using either *ESI* or *PSI* criteria) were compared with an averaged activity across all data points, recorded from the corresponding position bins and expressing the same map during the reference period with respective context identity. The data points with no eligible position-matched control values were excluded from the analysis.

#### Theta oscillations amplitude and frequency

The amplitude of theta oscillations was calculated from bandpassed LFP signal (6–11 Hz) using Hilbert transform and z-scored for whole session. The average theta amplitude was calculated for 2 seconds before and after each teleportation. To compare the theta amplitude across different network states, the average amplitude was calculated for”correct”, “incorrect” and “mixed” bins (20 theta bins post-teleportation interval) and then averaged across each session. The theta frequency was measured from the averaged duration of a theta bin for 20 bins before and after each teleportation.

#### Spatial coding

The correlation analysis of spatial coding was performed by Pearson momentary correlation between population vectors, consisting of spike numbers emitted by each recorded cell during the theta bin and the template population vector for a given spatial bin. The template population vectors for each bin were constructed from mean rate values for the corresponding bin, obtained from smoothed rate maps. The correlation coefficients were evaluated for examined theta cycles and compared with the averaged values of eligible template population vectors, performing Student’s t- test on the obtained values after Fischer z-transformation. The coding error was determined by correlating momentary population vectors with template vectors for all bins across the given environment, and corresponded to the distance between the bin with highest correlation and the real position of the rat.

To assess cell activity within its place field, smoothed template rate maps were normalized by their respective peak rates, obtaining values ranging from >  = 0 to 1, corresponding to the periphery and center of the place field, respectively. To evaluate the shift of activity towards the firing field periphery, we computed an averaged normalized firing rate value per activity event per theta cycle for examined bins. Increased activity within place field zones (the plot in Fig. 2d & Suppl. Fig. 5e) was quantified by first taking the distribution of normalized template values corresponding to activity events occurring during control theta cycles and computing 25^th^, 50^th^ and 75^th^ percentile which defined the respective zone borders. Here we considered only one randomly chosen theta cycle from all eligible template session controls for each examined post-teleportation bin. Thereafter, the number of activity events from post-teleportation bins with normalized reference firing rate values belonging to a given place field zone was divided by the corresponding number of activity events from the control data. Chi-square goodness-of-fit test was used to evaluate difference in distribution of all activity events from “correct” post-teleportation bins vs reference bins in “infield” and “outfield” zone, with border set to 25^th^ percentile (10.55% of peak firing rate) and expected distribution corresponding to reference data.

The positional information (*I*_*pos*_) was calculated for active cells as:

$${I}_{{pos}}={p}_{nx.}{\mathrm{log}}_{2}({p}_{nx}/{p}_{n})$$

where *p*_*nx*_ is the probability of the cell firing *n* spikes at position *x* and *p*_*n*_ is the probability of the cell firing *n* spikes in the given environment. The probabilities were calculated by approximating both firing at the given spatial bin and in the whole environment to be a Poisson process, with rates corresponding to mean template firing rates for the given bin and context, respectively. For each examined and control theta bin, average *I*_*pos*_ was calculated for active cells. The selection of bin specific activity for respective context was based on presence of nonzero template rate for respective bin.

#### Characteristics of the recruited cells

The mean firing rates were extracted from the activity during the template sessions. ESI values were positive for concordant specificity. The place field size was defined as a number of pixels in the template rate map with >50% of peak firing rate. The corresponding characteristics were calculated for each activity event occurring in the ‘outfield zone’ and the values were compared across the”correct” post-teleportation bins and reference data.

#### Place field centers distance

The distribution of distances between place field centers for cells simultaneously active at their respective place fields’ peripheries during a single theta cycle was compared to distribution of distances between field centers for all cell pairs of the given ensemble.

### Analysis of the mixed cycles

To calculate the proportion of mixed states, mixed bins were categorized according to the number of active cells into categories of bins containing from 2 to up to 8 cells. For each category, the relative proportion of the mixed bins to all bins with the same number of active cells was estimated. Thereafter, two-way ANOVA was performed to test the main effect of reference/post-teleportation condition with the effect of the number of active cells as the other independent variable. This approach was employed, as we expected the mixed state detection rate generally to increase with the number of active cells, and hyperactivity could contribute to the emergence of spurious detection of mixing.

The phase that was used to cut the recording to minimize the mixing of ensembles was obtained for each recording by repeatedly cutting the data at different phases of theta oscillations, with the corresponding phase shifted by 10 degrees for each of all 36 iterations. Phase values were determined by interpolating between peaks (0/360 degrees) and troughs (180 degrees) of LFP signal bandpassed for theta frequency (6–11 Hz).

The ensemble mix at 10 msec timescale was evaluated by cutting the data into fixed-length 10 msec bins and calculating number of mixed bins for 240 before and after cue switch for each teleportation.

The mixing at gamma cycle level: LFP signal was bandpassed for gamma frequency (25–100 Hz) and for each session, gamma phase with minimal spiking activity was extracted. This phase was then used for binning data into individual gamma bins. The number of mixed states was calculated for 100 gamma bins before and after cue switch for each teleportation.

The significance of ensemble separation was estimated by shuffling activity across post-teleportation population vectors and quantifying the number of mixed states for each of 10 000 randomizations. The analysis was restricted to theta cycles with at least 2 active cells, following the first correct bin after the teleportation, detected by ESI criteria. Only the teleportations where at least 3 such cycles were present, and which included at least 4 activity events of cells with strong specificity (abs(*ESI*) > 0.8) for each context, were considered.

#### Statistical analysis

The comparison of number of spikes, active cells, spikes per active cell, theta amplitude, theta wave length, interneuronal activity, mixing at short timescales and the change in running between pre-teleportation and the post-teleportation period was assessed by Wilcoxon signed-rank test over all teleportations. The activity level, speed, spatial coding error, change in activity within a place field and positional information during the “correct” and the “incorrect” theta bins were compared with the respective reference values by Wilcoxon signed-rank test over all the considered bins. The speed during the “correct” and the “incorrect” theta bins were compared by Wilcoxon rank-sum test across all examined bins. The characteristics of cells active during examined “correct” and reference data were compared by Wilcoxon rank-sum test across all activity events. The changes in correlation with the template population vector were assessed by paired-samples t-test after Fischer z-transformation over all the considered bins. The increase in the mixed states’ incidence was evaluated by two-way ANOVA, with the main effects of reference/post-tele condition and number of active cells per theta bin. Theta amplitude during different network states was computed for average values per recording day using one-way ANOVA. The change in distribution of activity in the ‘infield’ and the’outfield’ zones was evaluated by Chi-Square goodness-of-fit test. The place field center proximity for cell pairs active in the’outfield’ zone was evaluated by Kolmogorov-Smirnov test. Theta level ensemble separation was evaluated by randomization test. The results were considered significant if p < 0.05.

## Supplementary information

Retrieval of spatial representation on network level in hippocampal CA3 accompanied by overexpression and mixture of stored network patterns

## Data Availability

The dataset used in this publication is available on request at *karel.jezek@lfp.cuni.cz*.
