# Supplementary material for: Retrieval of spatial representation on network level in hippocampal CA3 accompanied by overexpression and mixture of stored network patterns
Source: Sci Rep. 2019 Aug 8;9:11512. doi: 10.1038/s41598-019-47842-w (PMC6687893; doi:10.1038/s41598-019-47842-w)
Supplement: Supplementary file 1 — Retrieval of spatial representation on network level in hippocampal CA3 accompanied by overexpression and mixture of stored network patterns [file 41598_2019_47842_MOESM1_ESM.pdf]

Retrieval of spatial representation on network level in hippocampal CA3 accompanied by overexpression and mixture of stored network patterns

Frantisek Zitricky and Karel Jezek

SUPPLEMENTARY MATERIALS

Supplementary Methods

Animals

Six adult Long Evans male rats were used throughout the study. After the surgery they were kept in individual cages with free access to water and food. The recovery lasted for 10 days, then the animals started to be gently food deprived so that their body weight did not drop below 85 % of their original weight.

Electrode preparation and surgery

Single unit neuronal activity was recorded in hippocampal subfield CA3. Rats were implanted with a custom made hyperdrive allowing an independent positioning of 14 tetrodes organized into a circular bundle. Tetrodes were twisted from 17 um insulated platinum-iridium wire (90% and 10%, respectively, California Fine Wire Company). Electrode tips were platinum plated to adjust their impedance to 120 – 250 kOhm (at 1 kHz).

Anesthesia was introduced by placing the rat into a plexiglass chambre with seal top filled with isoflurane vapour. Then the animal was injected with an intraperitoneal injection of Equithesin (pentobarbital and chloral hydrate in a dose of 1.0 ml per 250 g body weight). After the head was shaved, the animal was placed into the stereotaxic frame. Breathing, heart action and reflexes were monitored continuously. The hyperdrive was then implanted above the right dorsal hippocampus at coordinates AP 3.8 mm, DV 1.0 mm and ML 3.2 mm relative to bregma. Seven to nine stainless steel screws and dental acrylic were used to stabilize the implant on the skull. Two of the screws placed in the frontal bone served as the hyperdrive ground.

Tetrode positions

The tetrodes were slowly lowered in CA3 within 2-3 weeks after the surgery while the rat was resting in a comfortable pot outside of the recording chamber. The recording reference electrode was positioned in corpus callosum. Additional reference for EEG was placed in stratum lacunosum moleculare.

Recording procedures

Neural activity was recorded while the rat was exposed to a procedure described by Jezek et al. (2011, bellow). The signal was recorded differentially against the reference tetrode. The hyperdrive was connected to a multichannel, impedance matching, unity gain headstage and its output was conducted through a 82-channel commutator to a Neuralynx digital 64 channel data acquisition system.

The signal was band-pass filtered at 600 Hz–6 kHz. Unit waveforms above individually set thresholds (45-70 uV) were time-stamped and digitized at 32 kHz. The position of the light emitting diodes on the headstage was tracked at 50 Hz to assess the animal's spatial coordinates. For the purpose of this study only data from intervals when the rat's movement speed exceeded 5 cm/sec were used. Broadband EEG from each tetrode was recorded continuously at 2000 Hz.

Spike sorting and cell classification

Spikes were sorted manually using 3D graphical cluster-cutting software (SpikeSort, Neuralynx). The feature space consisted of three-dimensional projections of multidimensional waveform amplitudes and energies. The putative pyramidal units were classified as a place cell if their mean firing rate exceeded 0.1 Hz and their firing rate maps displayed coherence >0.6 and sparsity <0.4 for at least one template session. Autocorrelation and crosscorrelation functions were used as additional separation tools. Cells with mean firing frequency above 10 Hz and with the peak to through duration shorter than 0.3 milliseconds were classified as interneurons.

Behavioural apparatus and training

In brief, rats were trained to set distinct representations of two environments of the identical shape (60x60 cm, 50 cm high walls) that differed in visual cues on their walls and floor, respectively. Black curtains surrounded the apparatus to prevent the animal to see the rest of the room. In environment A the LEDs organized in a circle were placed bellow the translucent floor and an additional light was placed on one of the walls to polarize the box. Box B was illuminated by a 60-cm-long array of LEDs lining 40 cm of the upper edge of the wall opposite to the directional LED in A and 20cm of one of the adjacent walls. The same amount of LED units was used across both environments. There were no other lights available except the LEDs in the apparatus. The training had four stages (Suppl. Fig. 1).

Initially, the boxes were located next to each other, connected by a corridor (20x20cm, width x length) that allowed the rat to freely move between them at least in three trials (20 min each) in order to develop a different set of path integrator coordinates for each box. Trials were separated by 20 min breaks while the rat rested on a towel in a pedestal outside of the curtains (phase 1). Whereas the initial phase 1 was repeated for three subsequent days, the other phases took one day each. In phase 2, the passageway was removed and the animal explored the boxes individually on alternating trials (3 trials in each, 10 min breaks). The next day (phase 3), the boxes were replaced by another box of the same size, made of the same material and equipped with both sets of lights. Its position was box was alternating at the two original locations between the trials. When presented at the original position of box A, the set of LEDs defining A was lighted; when in the place of box B, the respective lights of B were switched on instead. Again, there were three trials in each environment on alternating occasions. In the final phase, the box was presented at a central location between the two original box positions. Again, the animal received two pairs of three alternating trials with each set of lights for two consecutive days. Across all stages, at the beginning of each trial, the rat was placed into the environment with the eyes gently covered by the experimenter's palm without any further disorientation. Between trials, the boxes were thoroughly cleaned and dried. On the test day, the rat was first exposed to each box configuration (A and B, respectively) for two 10 min reference recordings. In the third trial, the lights identifying given environment (for example, A) were switched instantaneously to the other configuration (for example, B). Such 'teleportations' were performed every subsequent 40–60 s for 10 min. Another two reference sessions were recorded afterwards. The animal rested for 10 min as usual between the trials. The experiment was repeated on the subsequent day after lowering the electrodes deeper into the CA3 if sufficient amplitudes from new cells could be recorded. Animal movement was motivated by small crumbles of cookies thrown into the box at 10–20 s intervals. Three flavors of food were used: vanilla, chocolate and cereals without any additional flavor. Vanilla and unflavored were offered in one light configuration, chocolate and unflavored in the other in a balanced way. During teleportation trials, only unflavored food was offered.

Histology

After the experiment was finished, the rat was overdosed with Equithesin and was perfused intracardially with saline followed by 4 % formaldehyde. Brain coronal sections (30 µm) were stained with cresyl violet. Traces of all 14 tetrode locations were identified. Each tip location was considered as the place in the section before the tissue damage became negligible. Only recordings from tetrodes with their tips in CA3 were used in this study.

Recording day	1	2	3	4	5	6	7	8	9	10	11
Correct	43	48	41	41	75	64	31	19	90	104	122
Incorrect	46	66	36	23	25	11	13	3	14	14	8
Mixed	90	81	55	34	52	63	12	6	6	61	27

Supplementary Table 1. Categorization of theta network states during post-teleportation period on individual recording days

Supplementary Figure 1

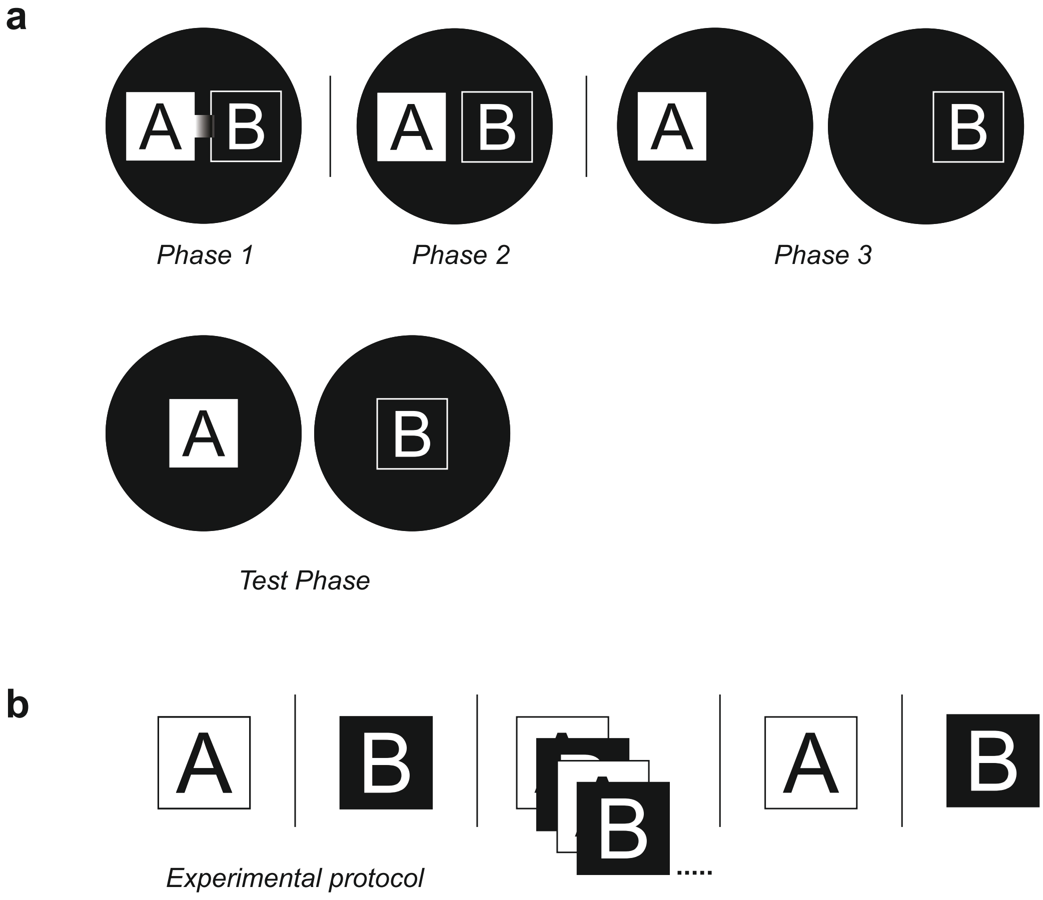

Supplementary figure 1

The rats were trained to explore two environments, each of identical box shape but defined by a specific configuration of light cues. a) During Phase 1, the rats moved freely between the boxes connected with a corridor to support formation of orthogonal place cell maps for each context. In Phase 2 the corridor was closed but both boxes retained their positions. In Phase 3 the rats explored a single box (equipped with both sets of light cues) with alternating context identity at the original locations. During the test phase, the box was placed in the middle between the original box locations. b) On the test day, the rats explored initially both contexts as in the final stage of the training (the reference sessions), followed by a teleportation session. The teleportation session started with light cues corresponding to one of the contexts and after 40-60 s of exploration the lights were instantaneously switched to the alternative configuration. The procedure was repeated each 40-60 s throughout the session. The teleportation session was followed by two other reference sessions, one for each of the environments.

Supplementary Figure 2

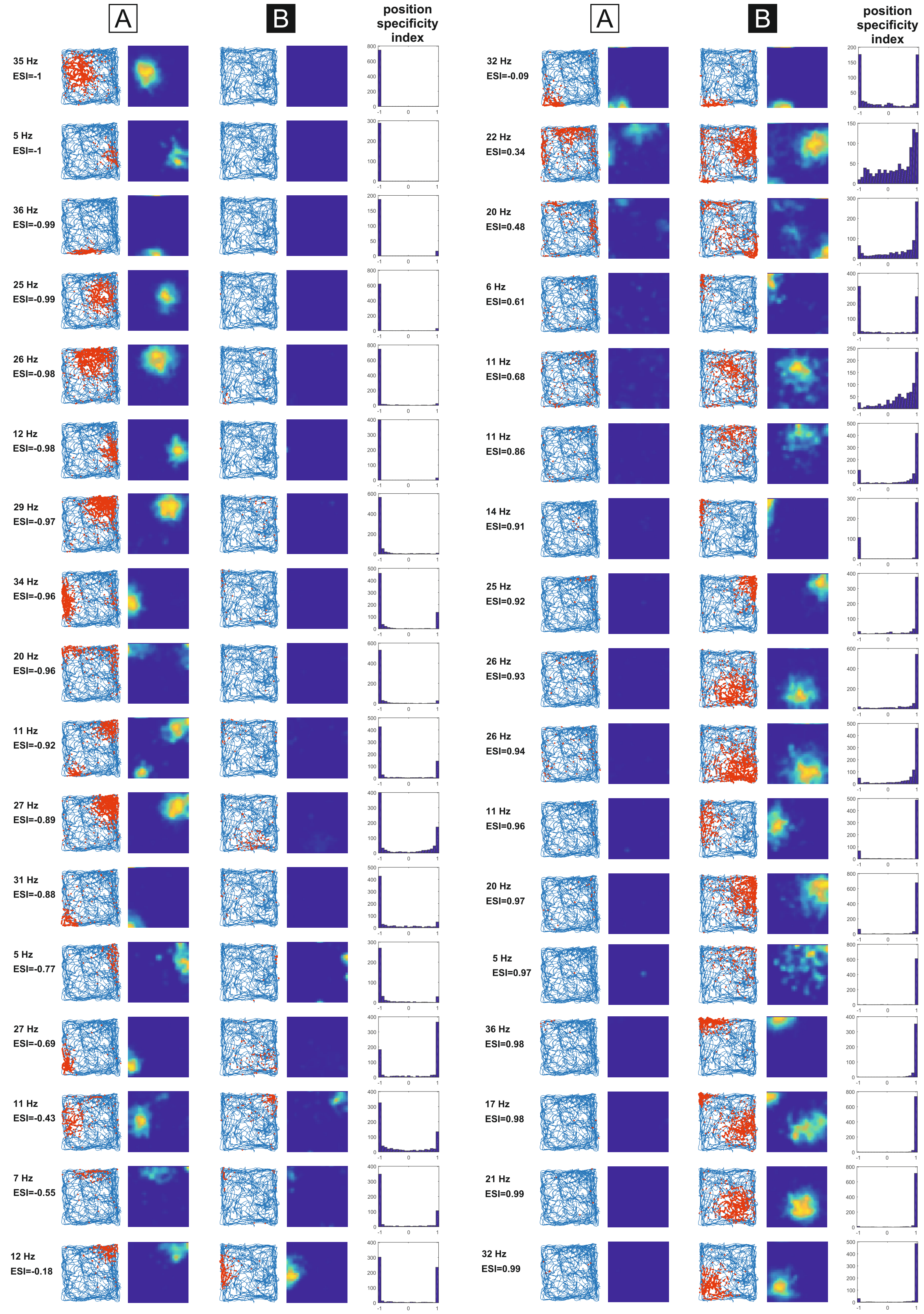

Supplementary figure 2
Example of cell population from rat no. 1. a) 34 cells were ranked according their respective ESI values. Peak frequency across both template sessions is reported together with the ESI value on the left. Scatter plots show individual spikes on top of the path traveled next to the respective rate map for both template sessions A and B, respectively. Distribution of all 30x30 position specificity index values is shown for each cell on the right.

Supplementary Figure 3

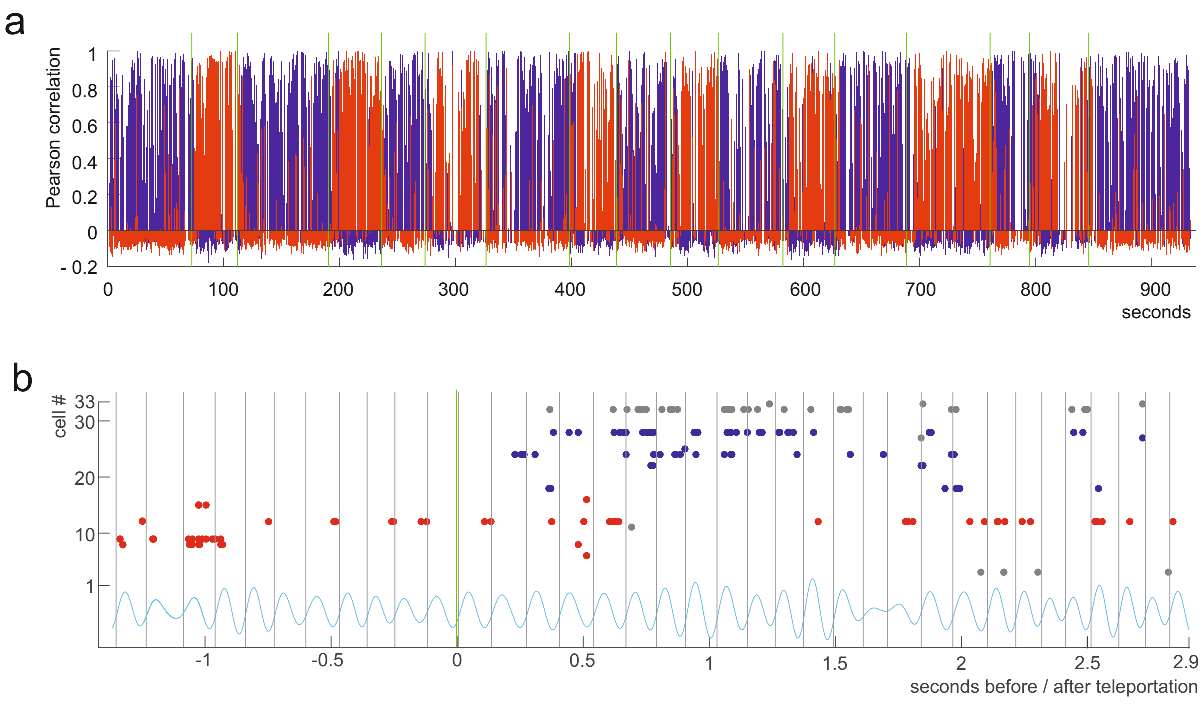

Supplementary figure 3

a) Example of evolution of population activity across the whole teleportation session from rats no. 1. The plot shows two arrays of correlation coefficients: between the data and the template A (red) and B (blue), respectively (as in Jezek et al., 2011). Green verticals mark moments of teleportation between environment A and B and vice versa.

b) Example of ensemble activity within the interval of -10 to 20 theta cycles before and after one of the teleportations depicted in a), respectively. Individual spiketimes from cells specific to the environment present before teleportation (ESIpre > 0.8) are in depicted in red, spiketimes from cells specific to the environment the animal was teleported to (ESIpost > 0.8) are in blue color. The grey circles mark activity of cells with lower specificity to either of the environments.

Supplementary Figure 4

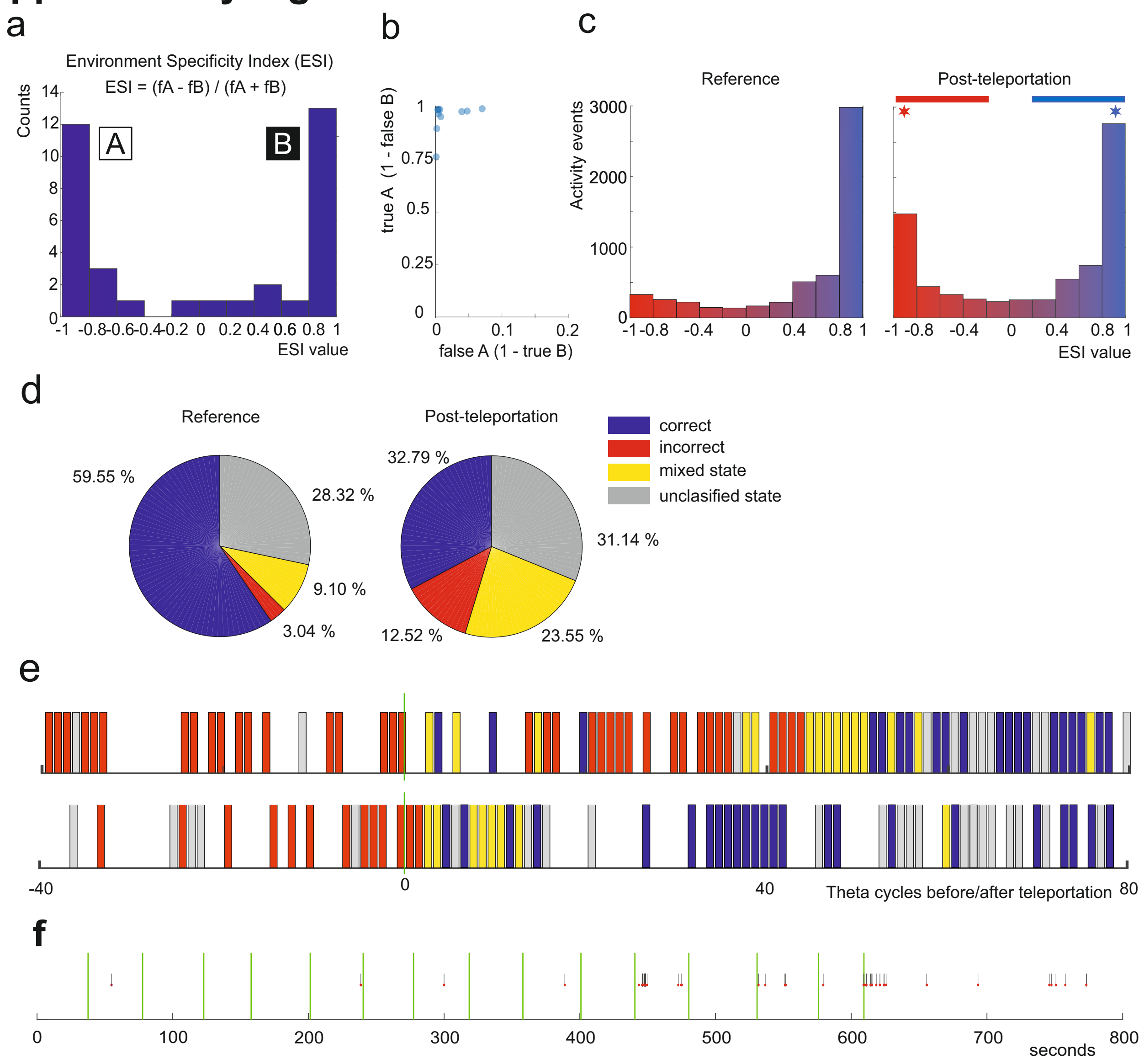

Supplementary figure 4

a) An example distribution of environment specificity index (ESI) values for one recording day. The active CA3 place cells tend to show firing preference for one of the environments.

b) Performance of ESI decoder on data from reference sessions per each recording day. Theta bins with detected expression of either map were classified based on their correspondence to the actual environment identity. The vertical axis represents the instances where during reference in context A, the map A was correctly decoded (true A = 1 – false B), the horizontal axis corresponds to instances where map A was decoded in context B (false A = 1 –true B).

c) Distribution of ESI values within 20 theta cycles before and after the cue switch. The positive values correspond to specificity for the current environment. The post-teleportation distribution displays marked increase in activity specific for the alternative context.

d) Distribution of decoded network states detected during the reference epochs of the teleportation session and the post-teleportation periods. During the 20 theta bins long post-teleportation period, there is a robust increase in the 'incorrect' and in the 'mixed' states, reflecting a competitive network dynamics.

e) An example of the evolution of population activity before and after a teleportation event classified according ESI. The cue switch (green bar) is followed by a transient instable period, where the network alternates between 'correct' (blue) and 'incorrect' (red) states. Considerable amount of theta bins were classified as 'mixed' (yellow), containing combined activity of cells highly specific for either of the environments. Grey bars represent unclassified bins with at least 2 active cells, the empty slots correspond to the bins with <2 active cells.

f) Evolution of coactivity (ISI <= 10 ms) incidence between cells exclusively active in either of the environments during initial template sessions. Prolonged recording after the last teleportation shows a long lasting coactivity formed as a response to repetitive teleportations (green lines).

Supplementary Figure 5

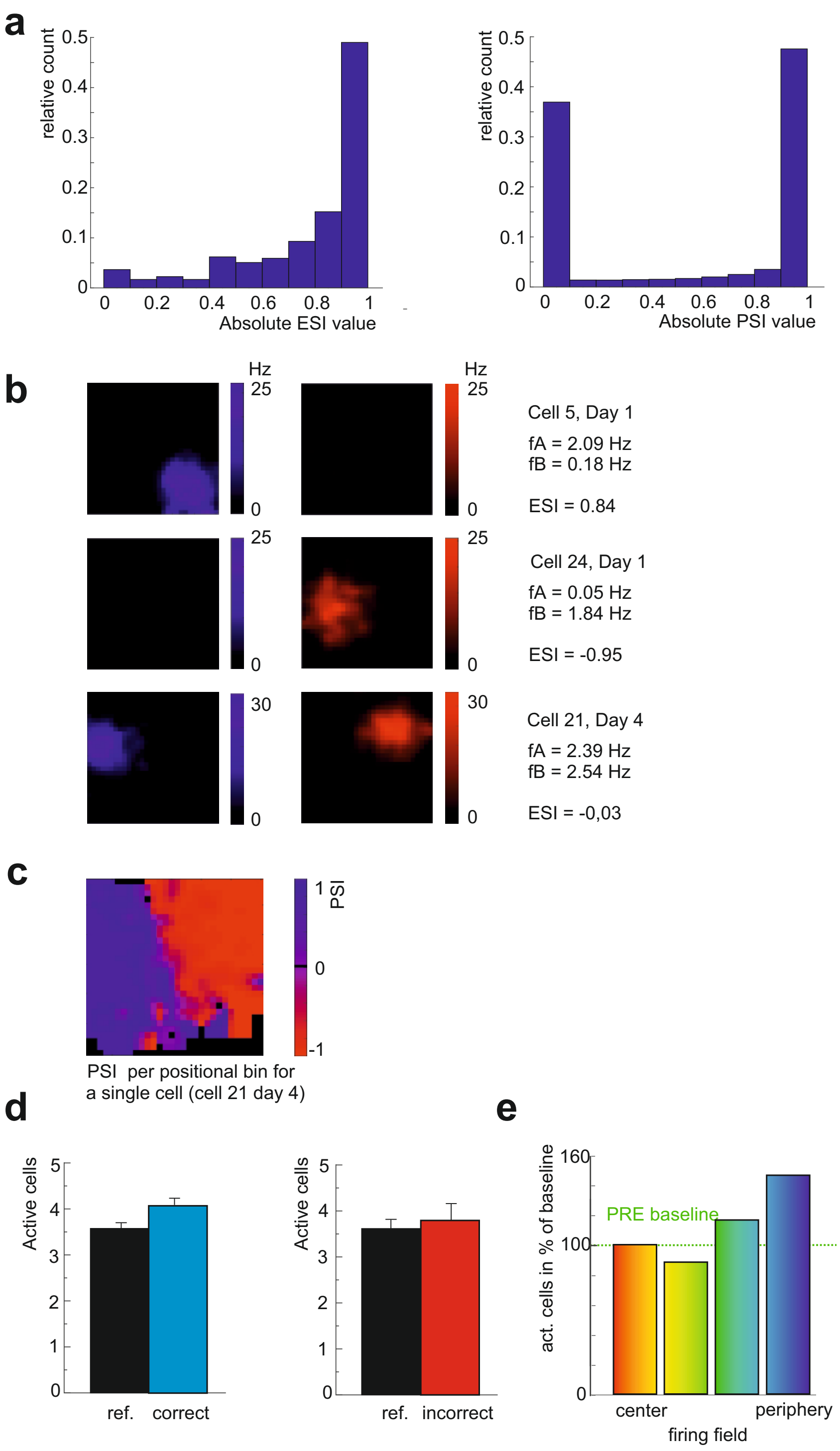

Supplementary figure 5

a) Distribution of absolute values of environment- (ESI, left) and position specific (PSI, right) indexes for all cells across all recordings.

b) The spatial ratemaps for both environments for three example cells with corresponding ESI values.

c) The PSI values across positional bins for cell no. 21 (Day 4) from the previous example.

d) The number of active cells during the 'correct' and the 'incorrect' states defined by PSI values for the active units. An increase in activity was observed during the 'correct' (3.56 ± 0.11 cells per TC reference, 4.07 ± 0.13 cells per TC post-tele, n = 153, Wilcoxon signed-rank test: z = 3.53, p = 4.1498e-04), but not during the 'incorrect' bins (3.08 ± 0.09 cells per theta cycle stable, 3.21 ± 0.13 cells per theta cycle post-tele, n = 71, Wilcoxon signed-rank test: z = 0.82, p = 0.4111).

e) Shift of activity towards the periphery of a firing field during expression of 'correct' state detected by PSI criteria (Wilcoxon signed-rank test: z=-4.20, p= 2.6477e-05).

Supplementary Figure 6

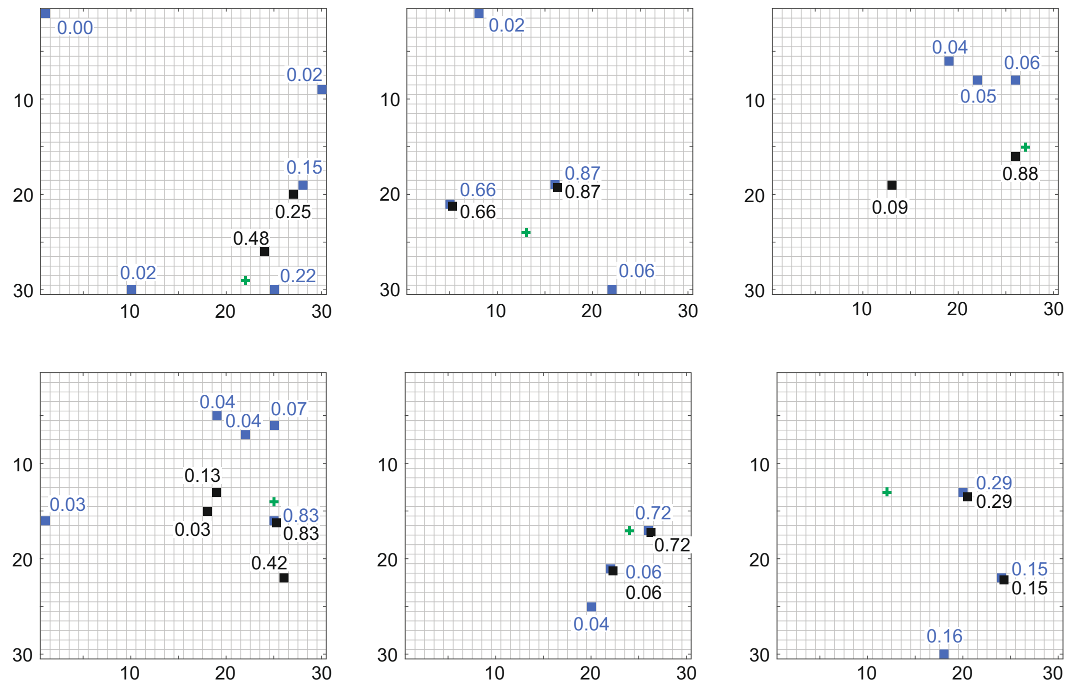

Supplementary figure 6

Examples of individual post-teleportation theta cycle-based population vectors. Each plot depicts positions of firing field centers of the cells active within a single theta cycle during the post-teleportation period (blue marks) and from the position-matched control (black marks). All the cases were categorized as 'correct' bins. Probability of firing at the actual position of the animal (green cross) is noted next to the marks.

Supplementary Figure 7

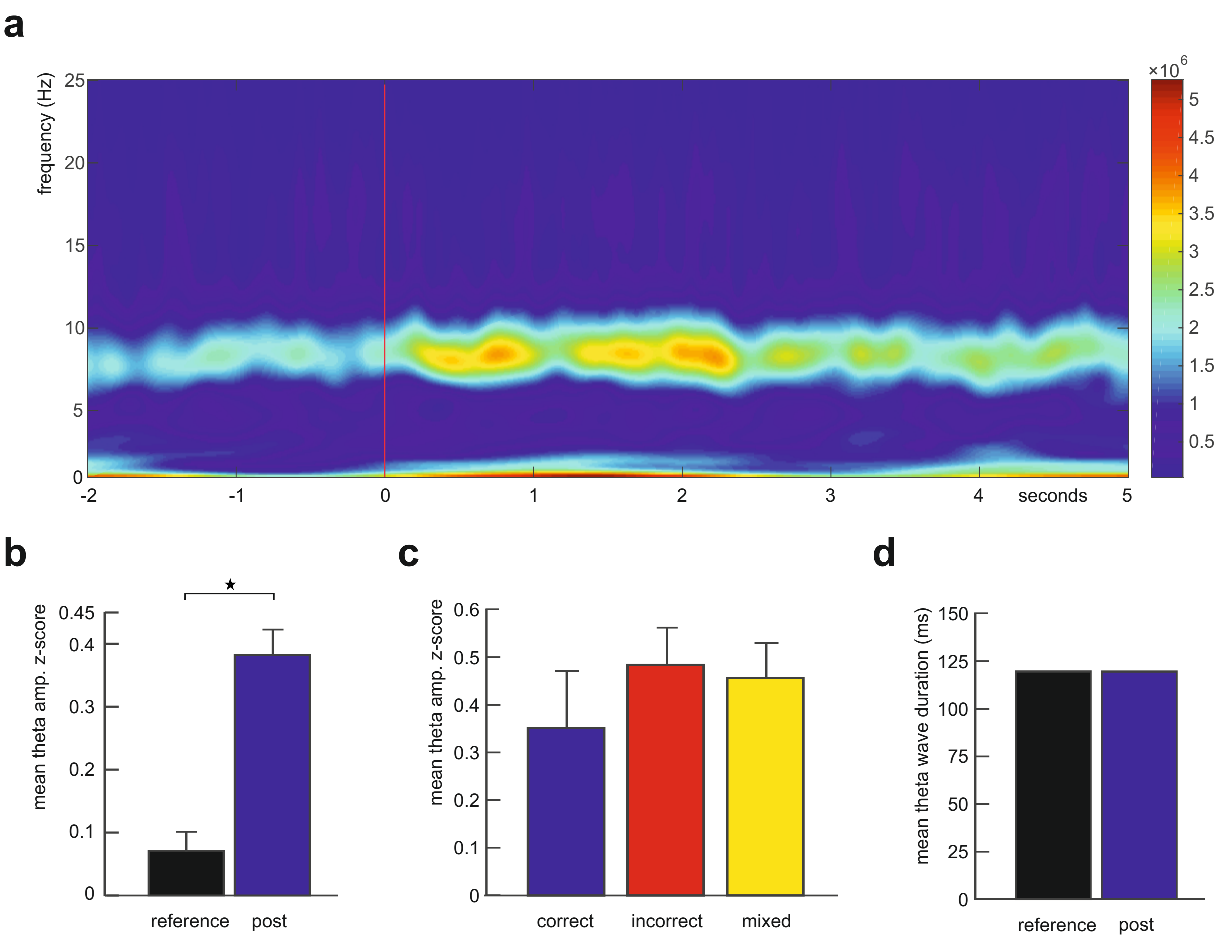

Supplementary figure 7

Analysis of theta oscillation before and after the teleportation vent

a) Example of time-frequency representation of local field potential (CA3 pyramidal layer) from interval -2 to +5 seconds before and after the telepoertation event from one experimental day (averaged across across 15 teleportations). Note the increase within the theta band (around 9 Hz) within the first two secons after the teleportation event.

b) Comparison of averaged z-score (± SEM) from theta frequency bandpassed (6-11 Hz) local field potential from intervals of two seconds before and after the teleportation event, respectively.

c) Averaged z-scores from postteleportation interval (as in c)) corresponding to an emergence of correct, incorrect and mixed population vectors.

d) Average theta wave duration during 2 seconds before and after the teleportation, respectively.

Supplementary Figure 8

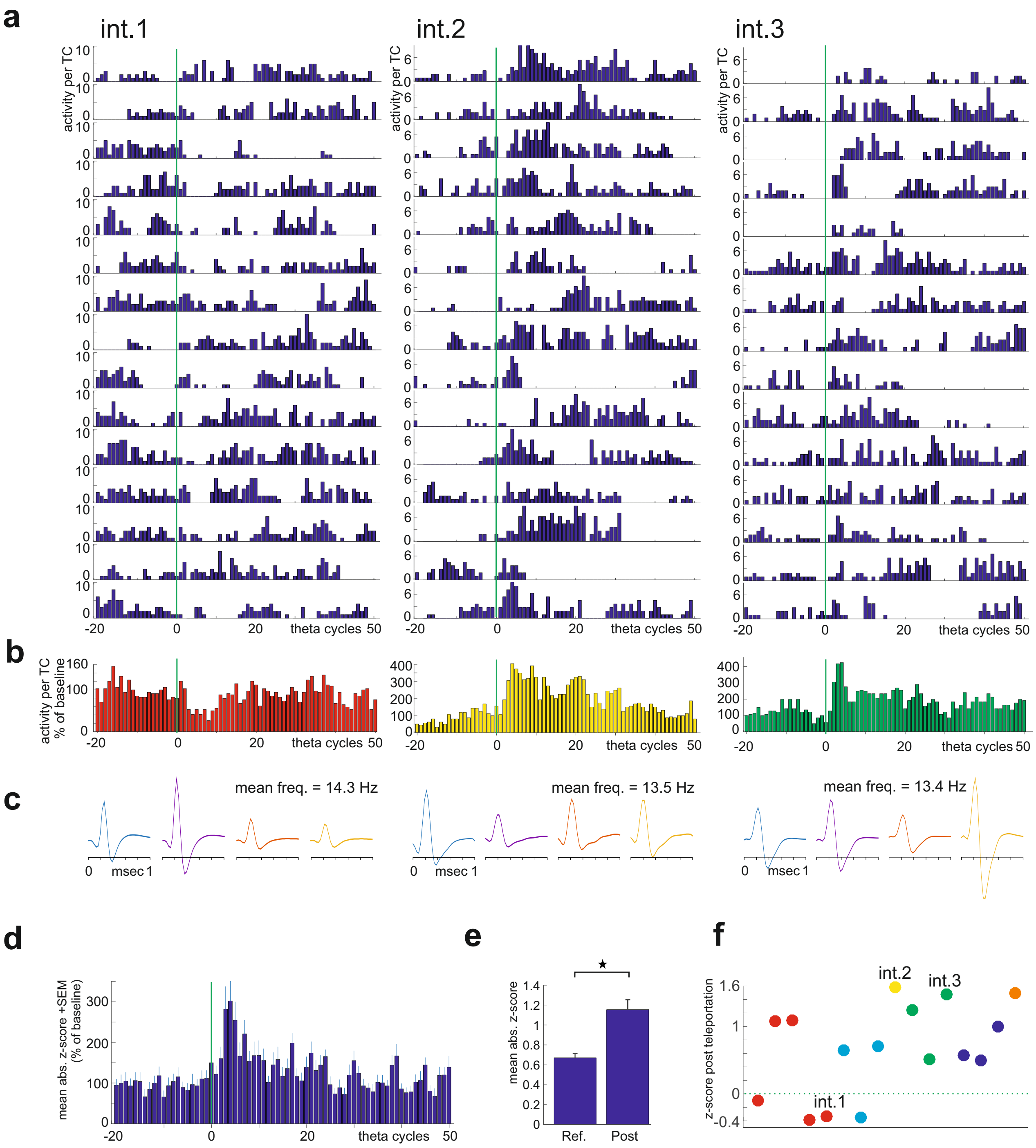

Supplementary figure 8

a-c) Examples of three interneurons recorded on independent experimental days. a) Activity of each interneuron across consecutive 15 teleportation trials (from top to bottom) during the period of -20 to +50 theta cycles before/after teleportation. b) Normalized average activity per theta cycle across all teleportation events. c) Wave-shape of each interneuron across four channels of a tetrode and the coresponding mean firing rate.

d) Evolution of averaged and normalized absolute z-score values (± SEM) of activity from 16 interneurons before and after all teleportation events in theta cycle temporal resolution.

e) Averaged absolute z-score values (± SEM) from periods of 20 theta cycles preceding and following the teleportation events, respectively.

f) Averaged z-score values of spiking activity after teleportation (20 theta cycles) across whole sample of interneurons. Same colors correspond to simultaneously recorded units. Colors and marks also correspond to the three interneuron examples from a) to c).
